# Dysregulation of Principal Circulating miRNAs in Non-human Primates Following Ischemic Stroke

**DOI:** 10.3389/fnins.2021.738576

**Published:** 2021-09-01

**Authors:** Jian Chen, Haiping Zhao, Yuyou Huang, Yuqian Li, Junfen Fan, Rongliang Wang, Ziping Han, Zhenhong Yang, Longfei Wu, Di Wu, Yumin Luo, Xunming Ji

**Affiliations:** ^1^Department of Neurosurgery, Institute of Cerebrovascular Diseases Research, Xuanwu Hospital, Capital Medical University, Beijing, China; ^2^National Clinical Research Center for Geriatric Disorders, Beijing, China; ^3^Beijing Institute for Brain Disorders, Beijing, China

**Keywords:** cerebral ischemia, rhesus monkeys, let-7g-5p, let-7g-3p_1ss22CT, plasma

## Abstract

Despite the recent interest in plasma microRNA (miRNA) biomarkers in acute ischemic stroke patients, there is limited knowledge about the miRNAs directly related to stroke itself due to the multiple complications in patients, which has hindered the research progress of biomarkers and therapeutic targets of ischemic stroke. Therefore, in this study, we compared the differentially expressed miRNA profiles in the plasma of three rhesus monkeys pre- and post-cerebral ischemia. After cerebral ischemia, Rfam sequence category revealed increased ribosomic RNA (rRNA) and decreased transfer RNAs (tRNAs) in plasma. Of the 2049 miRNAs detected after cerebral ischemia, 36 were upregulated, and 76 were downregulated (fold change ≥2.0, *P* < 0.05). For example, mml-miR-191-5p, miR-421, miR-409-5p, and let-7g-5p were found to be significantly overexpressed, whereas mml-miR-128a-5p_R − 2, miR-431_R − 1, and let-7g-3p_1ss22CT were significantly downregulated. Gene Ontology and Kyoto Encyclopedia of Genes and Genomes pathway analyses revealed that these differentially expressed miRNAs were implicated in the regulation of ubiquitin-mediated proteolysis and signaling pathways in cancer, glioma, chronic myeloid leukemia, and chemokine signaling. miRNA clustering analysis showed that mml-let-7g-5p and let-7g-3p_1ss22CT, which share three target genes [RB1-inducible coiled-coil 1 (RB1CC1), G-protein subunit γ 5 (GNG5), and chemokine (C-X-C motif) receptor 4 (CXCR4)], belong to one cluster, were altered in opposite directions following ischemia. These data suggest that circulating mml-let-7g may serve as a therapeutic target for ischemic stroke.

## Introduction

Circulating microRNAs (miRNAs) might be useful as surrogate biomarkers for the diagnosis or prognosis of pathological conditions such as acute stroke; finding early blood miRNA biomarkers to diagnose this disease, for instance, could drastically reduce treatment delays. In addition, emerging RNA therapeutics targeting mRNAs have already been approved by the Food and Drug Administration for clinical research (Erdos et al., 2021) and have gained attention in cerebrovascular research. For example, RNA-targeted therapies to lower Lipoprotein(a) are in clinical development. Currently, there are approximately 7680 patients with a history of myocardial infarction, ischemic stroke, and symptomatic peripheral arterial disease (Tsimikas et al., 2021); this number helps estimate the potential therapeutic relevance of miRNAs for the treatment of ischemic stroke.

Ischemic stroke is a complex disease with several risk factors. Despite the increasing focus on plasma miRNA biomarkers in acute ischemic stroke patients (Singh et al., 2021), there is limited knowledge about the differentially expressed miRNAs directly related to stroke itself, which has hindered biomarker development and the definition of therapeutic targets. In this study, we compared the differences in circulating miRNAs in non-human primates, namely rhesus monkeys, before and after cerebral ischemia to identify the miRNAs directly related to ischemic stroke. The results of this research could help bridge the gap between *in vivo* rodent models and human patients, leading to therapeutic applications and valuable and accurate information for early detection of ischemic stroke.

## Materials and Methods

### Animals

This study was approved by the Animal Use and Care Board of the Institute of Laboratory Animal Sciences, Capital Medical University. All experiments were performed in compliance with national guidelines and in accordance with the Guide for the Care and Use of Laboratory Animals. Three adult male rhesus monkeys (*Macaca mulatta*), aged 7–11 years and weighing 7.2–10.6 kg, were used in this study.

### Anesthesia

Animals were fasted for 12 h prior to the induction of anesthesia. Anesthesia was induced with ketamine (10 mg/kg, IM) and maintained intravenously with propofol (300 μg/kg/min). Eighteen-gage peripheral venous catheters were placed. Ventilation was controlled (Aridyne 3600; Graham, NC, United States). Intermittent positive pressure ventilation was performed for monkeys with a fixed respiratory rate. Non-invasive blood pressure, electrocardiogram, heart rate, oxygen saturation, blood gas, and rectal temperature were monitored (Wu et al., 2020a,b,c).

### Endovascular Surgery

As described previously by us in NHPs models, a Prowler-10 micro-catheter (Codman) with a SilverSpeedTM-10 Hydrophilic micro-wire was introduced into the guiding catheter and navigated to the distal end of M1 segment of the right MCA. Next, the clot was transferred into micro-catheter and flushed into the end of M1 segment with 2 mL saline. Based on cerebral angiography at 3 h post ischemia, we only included 3 monkeys with a still occluded M1-segment of MCA. Blood samples were drawn from the saphenous vein at baseline and 3 h after stroke onset (Wang et al., 2016; Wu et al., 2021). MRI scanning was performed on a Magnetom Trio MRI Scanner (3.0 T; Siemens AG, Siemens Medical Solutions, Erlangen, Germany). MRI sequences and parameters were reported in our previous study (Wang et al., 2016; Wu et al., 2021). Based on recent reports, DWI images obtained 1 day after ischemia were defined as “originally abnormal region” (Nagaraja et al., 2020).

### Small RNA Sequencing

Circulating miRNA was isolated using 500 μl of plasma. The experimental process was carried out according to the standard steps provided by Illumina (Hangzhou Lianchuan Biological Technology Co., Ltd., China), including library preparation and sequencing experiments. A Truseq small RNA sample prep kit (Illumina, San Diego, CA, United States) was used to prepare the small RNA sequencing library. After library preparation, the constructed library was sequenced using Illumina HiSeq 2000/2500, and the reading length was 1 × 50 bp. We determined the transcript profile for the plasma by RNA-seq with a median sequencing depth of 10 million mapped reads per sample (*n* = 3). The miRNA data analysis software provided by Lianchuan Biology was ACGT101-miR (LC Sciences, Houston, TX, United States). The analysis process was as follows: (1) quality filtering: the raw reads obtained by sequencing contain low-quality reads with adapters. In order to ensure the quality of information analysis, the raw reads must be processed to obtain clean reads. The data processing steps are as follows: (a) remove *N* (*N* means that the base information cannot be determined) is greater than 10% of reads; (b) remove the reads contaminated with the 5′ connector; (c) remove reads without 3′ linker sequence and insert; (d) remove polyA/T/G/C reads (most of the continuous polyA/T/G/C may be due to sequencing errors, and the information entropy is low, so analysis is not necessary). Linker information for Small RNA sequencing: 5′ connector: 5′-GTTCAGAGTTCTACAGTCCGACGATC-3′, 3′ connector: 5′-TGGAATTCTCG GGTGCCAAGG-3′; here we removed the data with very low expression levels from the differential miRNA expression (including the case where three samples are all zero or two are zero), which is only shown in Supplementary Data; (2) length screening: retain 18–26 nucleotide (nt) base length sequences; (3) RNA database alignment analysis: Rfam is a non-coding RNA (ncRNA) family database, including ribosomic RNA (rRNA), transfer RNA (tRNA), snoRNA, snRNA, miRNA, and other ncRNAs. We select the Rfam database to annotate the small RNA sequences obtained by sequencing, and find and remove the possible rRNA, snoRNA, snRNA, tRNA, and other non-miRNA sequences as far as possible; (4) miRNA identification: after effective data were obtained, the precursor and genome were compared for miRNA identification; the *t*-test test was used in this analysis. For the analysis of samples with biological duplicates, the threshold of *P* ≤ 0.05 was used to screen differentially expressed genes (Han et al., 2019; Jeon et al., 2019; Li et al., 2019); (5) identification of differentially expressed miRNAs; and (6) prediction of differential miRNA target genes. The data presented in the study are deposited in the GEO respository (https://www.ncbi.nlm.nih.gov/geo/query/acc.cgi?acc=GSE182429).

### Target Gene Prediction of Differential miRNAs

TargetScan (V5.0) and Miranda (v3.3a) were used to predict the target genes of significantly different miRNAs. The target genes predicted by the two software programs were screened according to the scoring criteria in each software program. In the TargetScan algorithm, target genes with a context score percentage <50 are removed, while in the Miranda algorithm, target genes whose maximum energy is ≥10 are removed (score ≥50, Miranda energy = 10). Finally, the intersection data of the two software packages was considered as the final target genes of the differential miRNAs, providing the Gene ontology (GO) and Kyoto Encyclopedia of Genes and Genomes (KEGG) annotation information.

### Enrichment Analysis of Differential miRNA Target Genes

Enrichment analysis mainly included two parts: GO function and KEGG path function annotation. First, the number of target genes corresponding to all selected miRNAs corresponding to each function or pathway annotation was counted, and then a hypergeometric test was applied to determine the mRNA of target genes corresponding to all selected miRNAs and GO/KEGG in the annotation library, and compared with the number of genes in the pathway (all genes with functional annotation, or all miRNA target genes with functional annotation). A *P*-value ≤ 0.05 was used as threshold for significance, and the function meeting this condition was defined as a function with significant enrichment in miRNA–mRNA relationship pairs. The main biological functions of miRNA–mRNA pairs can be determined by functional significance enrichment analysis. The significant enrichment analysis of pathways uses the KEGG pathway as a unit and applies a hypergeometric test to determine significantly enriched paths in the significant differentially expressed genes compared with the whole genomic background.

### Conservation of the Identified miRNA With Other Species

MicroRNAs is highly conserved among species. To identify the conserved rhesus monkeys miRNAs, we first collected the miRNA sequences from miRBase release 20.0 for selected species: Age, *Archangium gephyra*; aja, *Amycolatopsis japonica*; bta, *Bos taurus* (cattle); cfa, *Canis lupus familiaris* (dog); cgr, *Campylobacter jejuni* RM1221; chi, *Chlamydia psittaci* 02DC15; cja, *Cellvibrio japonicus*; cpo, *Coprothermobacter proteolyticus*; dma, *Desulfovibrio magneticus*; dno, *Dichelobacter nodosus*; eca, *Pectobacterium atrosepticum* SCRI1043; efu, *Enterococcus faecium* DO; ggo, *Gorilla gorilla gorilla* (western lowland gorilla); has, *Homo sapiens*; lca, *Lactobacillus paracasei* ATCC 334; lla, *Lactococcus lactis* subsp. lactis Il1403; mdo, *Monodelphis domestica* (opossum); mml, *M. mulatta* (rhesus monkey); mmr, *Maricaulis maris*; mmu: *Mus musculus* (house mouse); mne, *Mycobacterium neoaurum*; nle, *Nomascus leucogenys* (northern white-cheeked gibbon); oan, *Ornithorhynchus anatinus* (platypus); oar, *Octadecabacter arcticus*; ocu, *Oryctolagus cuniculus* (rabbit); oga, pal, pbl, *Paracoccidioides lutzii* Pb01; pha, *Pseudoalteromonas haloplanktis*; ppa, *Pan paniscus* (pygmy chimpanzee); ppy, *Pongo abelii* (Sumatran orangutan) (*Pongo pygmaeus abelii*); ptr, *Pan troglodytes* (chimpanzee); rno, *Rattus norvegicus* (rat); sbo, *Shigella boydii* Sb227; sha, *Staphylococcus haemolyticus* JCSC1435; sla, *Serpula lacrymans*; ssc, *Sus scrofa* (pig); ssy, *Sphingobium* sp. SYK-6; tch, and *Chlamydia trachomatis* F/11-96. The BLASTN program was used to compare the distributions of the miRNAs across the species. We further analyzed the conservation of miRNAs in selected species, and counted the frequency of miRNAs reported in this species in other species, and explored the existence of miRNA family in other species.

### Seed-Sequence-Based miRNA Families

In animals, miRNA and target genes inhibit mRNA expression mainly through incomplete complementary pairing. The Watson Crick pairing between the so-called seed sequence (second-7 nt) at the 5′ end of miRNA and the 3′ UTR of target gene is the most important factor for all miRNA target gene prediction.

## Results

### Biological Replication and Rfam Sequence Category of Rhesus Monkey Plasma miRNAs Libraries Before and After Cerebral Ischemia

**FIGURE 1:**
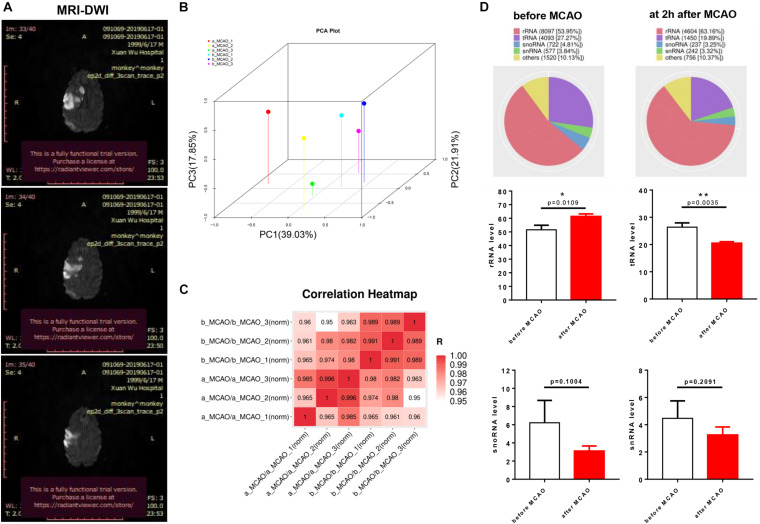
Biological replication and Rfam sequence category of plasma miRNAs libraries of rhesus monkey before and after cerebral ischemia. **(A)** Magnetic resonance imaging with diffusion-weighted imaging (MRI-DWI) (1 day) identified infarct site in models. **(B)** Principle component analysis (PCA) of plasma of rhesus monkey before cerebral ischemia compared with after cerebral ischemia. **(C)** Pearson correlation coefficients between samples using genes of *P* < 0.05. **(D)** Rfam classification among the other non-coding RNAs (e.g., rRNAs, tRNAs, snRNAs, and snoRNAs). b_MCAO_1-3, before ischemia; a_MCAO_1-3, after ischemia. *N* = 3. *p < 0.05 and **p < 0.01.

To evaluate the success of MCAO models, we found notable infarct sizes in the middle cerebral artery-supplied regions based on DWI images at 1 day after ischemia (Figure 1A). Principal component analysis using three principal components, which describe 78.79% of the variance, showed miRNAs to be significantly altered across the two groups (Figures 1A,B). Pearson correlation coefficients between samples showed that b_MCAO_1 (before ischemia) was similar to b_MCAO_2 (*r* = 0.966), and a_MCAO_1 (after ischemia) to a_MCAO_3 (*r* = 0.963) prior to a_MCAO_2 (*r* = 0.797) (Figure 1C). The length distribution of the filtered valid data was calculated based on the original sequencing data. Most reads were 18- to 26-nt long with a peak around 22 nt, demonstrating that the small RNA libraries were highly enriched in mature miRNAs. The length distribution of the identified miRNAs was consistent with the canonical size range of mammalian miRNAs, confirming the reliability of our small RNA-seq results. Genomic mapping of these reads showed that the predominant RNA species (based on read counts) in both libraries were miRNAs. Rfam classification among the other ncRNA (e.g., rRNAs, tRNAs, snRNAs, and snoRNAs), comprising <2% of total reads, rRNA was increased, and tRNA decreased after cerebral ischemia (*P* < 0.05, Figure 1D).

### Differentially Expressed Rhesus Monkey Plasma miRNAs Before and After Cerebral Ischemia

**FIGURE 2:**
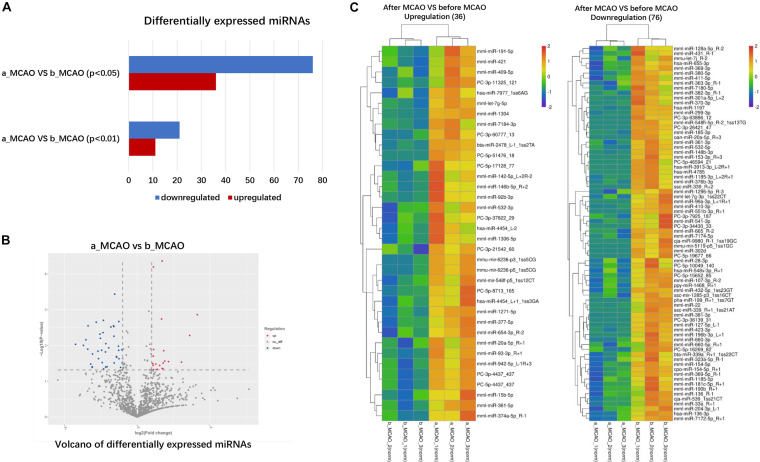
Differentially expressed plasma miRNAs of rhesus monkey before and after cerebral ischemia. **(A)** The blue represents down–down miRNA, and the number represents up–down miRNA. **(B)** Volcano plot analysis showing differentially expressed miRNAs, using log_2_ (fold change) as abscissa and –log_10_ (*P*-value) as ordinate. The red and blue dot represent the up-regulated or down-regulated differential expression genes, and the gray dot represents the non-significant differential expression genes. **(C)** Hierarchical clustering heatmap analysis of upregulated and downregulated miRNAs detected in plasma of rhesus monkey before and after cerebral ischemia. Red indicates high expression genes and blue indicates low expression genes. b_MCAO_1-3, before ischemia; a_MCAO_1-3, after ischemia. *N* = 3.

The frequency of differentially expressed genes was determined pre- and post-cerebral ischemia. Of the 2049 miRNAs detected, 36 were upregulated (Table 1) and 76 downregulated (Table 2) after cerebral ischemia (fold change ≥2.0, *P* < 0.05) (Figure 2A). The overall distribution of differentially expressed miRNAs is presented in Figure 2B. Hierarchical clustering of upregulated and downregulated miRNAs showed differences before and after cerebral ischemia (Pang et al., 2019; Zhang L. et al., 2019; Figure 2C). mml-miR-191-5p, mml-miR-421, mml-miR-409-5p, and mml-let-7g-5p were significantly upregulated, whereas mml-miR-128a-5p_R − 2, mml-miR-431_R − 1, and mml-let-7g-3p_1ss22CT were significantly downregulated in rhesus monkeys following cerebral ischemia.

**TABLE 1:** Summary information of upregulated miRs.

Name	log_2_ (fold change)	*P*-value (*t* test)	Expression level
bta-miR-2478_L − 1_1ss2TA	1.72	0.0000	Middle
mml-miR-1304	1.12	0.0001	Middle
mml-let-7g-5p	0.80	0.0011	High
mmu-mir-6236-p5_1ss5CG	4.14	0.0014	Middle
mmu-mir-6236-p3_1ss5CG	4.14	0.0014	Middle
mml-miR-1271-5p	1.68	0.0018	High
PC-3p-4437_437	0.99	0.0030	Middle
PC-5p-4437_437	0.99	0.0030	Middle
PC-3p-60777_13	3.61	0.0052	Middle
mml-miR-942-5p_L − 1R + 3	0.88	0.0093	Middle
mml-miR-361-5p	0.64	0.0105	High
mml-miR-377-5p	0.91	0.0124	Middle
mml-miR-93-3p_R + 1	1.13	0.0129	Middle
mml-mir-548f-p5_1ss12CT	1.83	0.0140	Middle
mml-miR-654-3p_R − 2	0.96	0.0171	High
mml-miR-374a-5p_R − 1	1.11	0.0198	Middle
mml-miR-20a-5p_R + 1	0.52	0.0254	High
mml-miR-15b-5p	0.71	0.0259	High
mml-miR-421	0.69	0.0269	Middle
mml-miR-7184-3p	1.90	0.0274	Middle
hsa-miR-4454_L + 1_1ss3GA	2.15	0.0283	Middle
PC-5p-8713_165	3.05	0.0292	Middle
PC-5p-17128_77	1.78	0.0308	Middle
mml-miR-532-3p	1.07	0.0319	Middle
mml-miR-1306-5p	1.29	0.0330	Middle
mml-miR-409-5p	1.10	0.0360	High
hsa-miR-4454_L − 2	1.54	0.0362	Middle
hsa-miR-7977_1ss6AG	1.72	0.0438	Middle
PC-3p-37822_29	1.77	0.0446	Low
mml-miR-191-5p	0.98	0.0459	High
mml-miR-142-5p_L + 2R − 2	1.39	0.0465	High
PC-3p-11325_121	1.12	0.0471	Middle
PC-3p-21542_60	1.31	0.0474	Middle
mml-miR-92b-3p	0.85	0.0479	Middle
mml-miR-146b-5p_R + 2	0.94	0.0488	Middle

*The normalized expression data is sorted from low to high, middle is the copy number as long as one sample is higher than 10 and the copy number in all samples is less than the average (sum of copy numbers)/(number of samples × total number of miRNAs) miRNA; high is the miRNA whose copy number is greater than the average in a sample.*

**TABLE 2:** Summary information of downregulated miRs.

Name of downregulated miRs	log_2_ (fold change)	*P*-value (*t* test)	Expression level
hsa-miR-1197	−1.54	0.0004	Middle
mml-miR-299-3p	−2.32	0.0020	High
mml-miR-532-5p	−1.26	0.0021	High
mml-miR-7174-5p	−2.81	0.0022	Middle
mml-miR-423-3p	−1.63	0.0028	High
PC-5p-15652_85	−3.38	0.0028	Middle
mml-miR-154-5p	−1.60	0.0030	Middle
mml-miR-380-5p	−1.81	0.0030	Middle
mml-miR-361-3p	−0.73	0.0039	Middle
cpo-miR-154-5p_R + 1	−2.34	0.0044	Middle
mml-miR-665_R − 2	−0.65	0.0044	Middle
mml-miR-185-3p	−2.56	0.0048	Middle
ssc-mir-1285-p3_1ss16CT	−1.60	0.0055	Middle
mml-miR-410-3p	−2.53	0.0063	Middle
mml-miR-381-3p	−3.53	0.0069	High
mml-miR-7172-5p_R + 1	−2.16	0.0089	Middle
mml-miR-376b-3p	−4.24	0.0093	Middle
mml-miR-660-3p	−3.69	0.0099	Middle
hsa-miR-136-3p	−1.29	0.0105	Middle
mml-miR-107-3p_R − 2	−0.59	0.0110	Middle
mml-miR-148b-3p	−1.95	0.0116	High
mml-miR-369-5p_R − 1	−0.94	0.0125	Middle
mml-miR-127-5p_L − 1	−3.40	0.0127	Middle
mml-miR-369-3p	−1.05	0.0131	Middle
bta-miR-339a_R + 1_1ss22CT	−2.45	0.0135	Middle
hsa-miR-655-3p	−1.27	0.0136	Middle
ppy-miR-1468_R + 1	−2.70	0.0144	Middle
mml-miR-1185-3p_L + 2R + 1	−2.18	0.0152	Middle
mml-let-7g-3p_1ss22CT	−3.07	0.0173	Middle
mml-miR-432-5p_1ss23GT	−0.75	0.0185	High
mml-miR-181c-5p_R + 1	−1.42	0.0202	Middle
cja-miR-539_1ss21CT	−1.42	0.0214	Middle
mml-miR-382-3p_R − 1	−1.00	0.0232	Middle
mmu-let-7j_R − 2	−2.66	0.0233	Middle
mml-miR-204-3p_L − 1	−1.25	0.0234	Middle
hsa-miR-548x-3p_R + 1	−2.40	0.0301	Middle
mml-miR-128a-5p_R − 2	−1.03	0.0308	Middle
mml-miR-301a-5p_L + 2	−1.58	0.0308	Middle
mml-miR-1296-5p_R − 3	−0.90	0.0313	Middle
mml-miR-411-5p	−0.86	0.0325	High
PC-5p-10049_140	−3.19	0.0330	Middle
mml-miR-7180-5p	−0.87	0.0336	Middle
mml-miR-1185-5p	−0.98	0.0347	Middle
mml-miR-28-3p	−0.71	0.0361	High
mml-miR-363-3p_R − 1	−0.46	0.0365	Middle
PC-5p-16269_82	−3.10	0.0393	Middle
mml-miR-22	−2.07	0.0393	High
mml-miR-541-3p	−2.17	0.0401	Middle
mml-miR-323a-5p_R − 1	−0.78	0.0405	Middle
mml-miR-33a_R + 1	−3.09	0.0409	Middle
mml-miR-136_R − 1	−1.14	0.0411	Middle
mml-miR-431_R − 1	−0.92	0.0412	Middle
mml-miR-190b_R + 1	−0.75	0.0428	Middle
PC-3p-7925_187	−0.97	0.0441	Middle
mml-miR-370-3p	−2.31	0.0455	High
mml-miR-660-5p_R + 1	−1.02	0.0483	Middle

*The normalized expression data is sorted from low to high, middle is the copy number as long as one sample is higher than 10 and the copy number in all samples is less than the average (sum of copy numbers)/(number of samples × total number of miRNAs) miRNA; high is the miRNA whose copy number is greater than the average in a sample.*

### Enrichment Analysis of GO and KEGG Pathways According to the Target Genes of Differentially Expressed miRNAs

**FIGURE 3:**
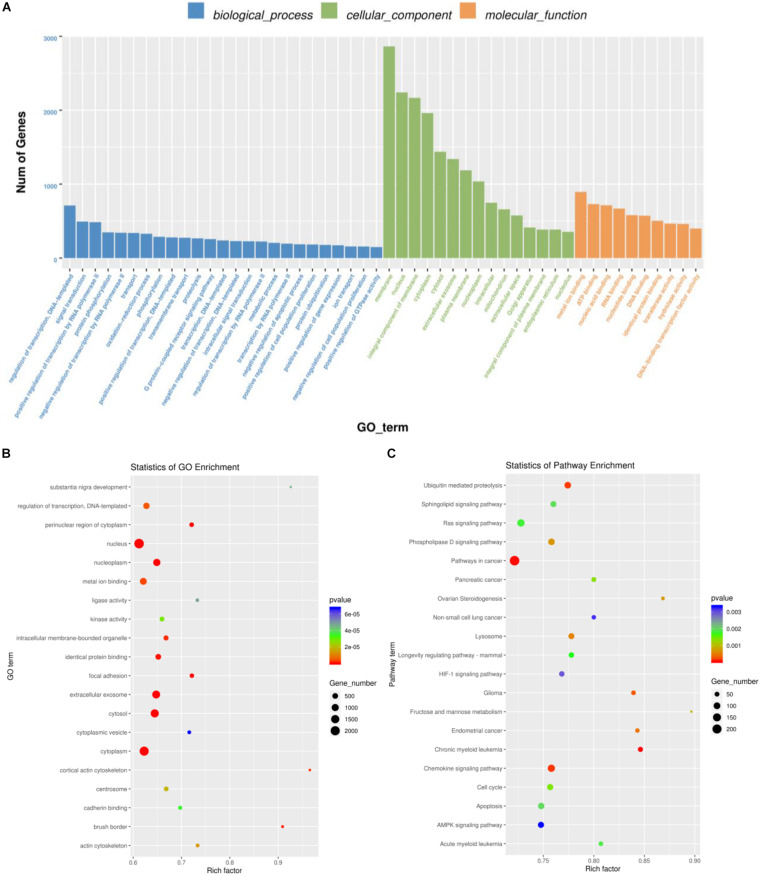
Enrichment analysis of GO and KEGG pathways according to the target genes of differentially expressed miRNAs. **(A)** Top enriched terms of biological process, cellular component, and molecular function category in Gene Ontology enrichment analysis. The abscissa is the classification of GO, and the ordinate is the percentage of target genes. **(B)** Top 20 significantly enriched GO pathways of differentially expressed miRNAs-targeted genes. **(C)** Top 20 significantly enriched KEGG pathways of differentially expressed miRNAs-targeted genes. GO, Gene ontology; KEGG, Kyoto Encyclopedia of Genes and Genomes database.

In all the differentially expressed miRNA target genes, we annotated the GO terms of these genes according to molecular function, cellular components, and biological processes, and the GO functions in each category were ranked from high to low according to the number of target genes. Regulation of transcription, DNA-templated, membrane, and metal ion binding was the most changed number of genes, respectively, in biological processes, cellular components, and molecular function (Figure 3A). GO enrichment analysis where the rich factor represents the number of GO differential genes/the total number of GO genes, revealed that differentially expressed miRNAs were significantly involved in the regulation of signaling pathways, including those involving the nucleus, extracellular exosome, and cytoplasm (*P* < 0.001, Figure 3B). The results of KEGG enrichment analysis by ggplot2 are shown by scatter plot: rich factor represents the number of different genes located in the KEGG/the total number of genes located in the KEGG, revealed that differentially expressed miRNAs were significantly involved in the regulation of signaling pathways including pathways in cancer, and chronic myeloid leukemia pathways; and chemokine signaling pathways (*P* < 0.001, Figure 3C).

### Clustering Analysis of miRNA Expression

**FIGURE 4:**
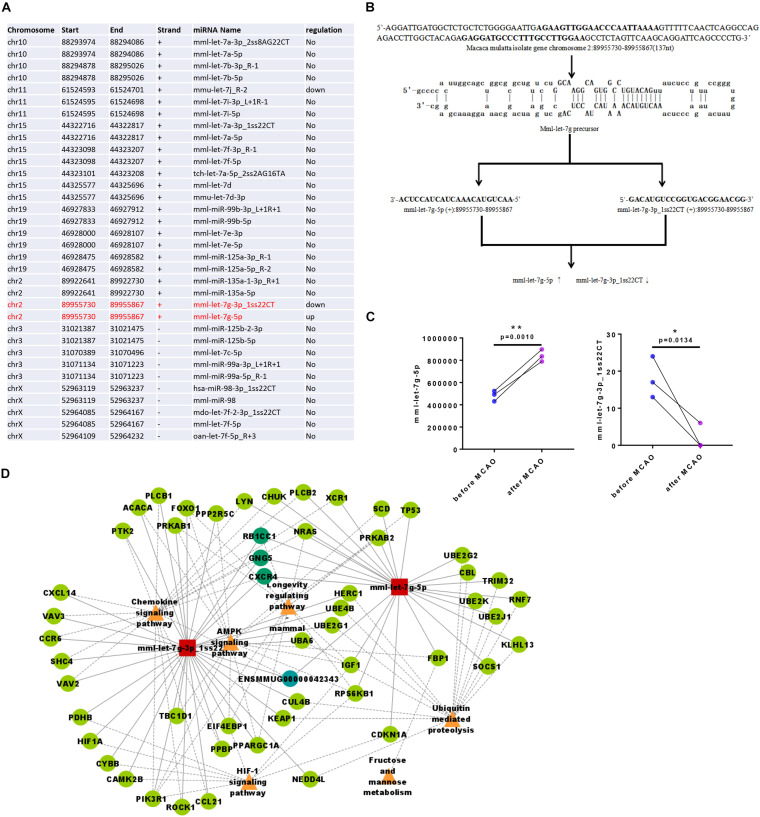
Clustering analysis of miRNA expression of rhesus monkey. **(A)** mml-let-7g-5p and mml-let-7g-3p_1ss22CT belongs to one cluster. **(B)** The stem-loop structures of the two pre-miRNAs that can yield mature miRNA sequences (mml-let-7g-5p and mml-let-7g-3p_1ss22CT). **(C)** mml-let-7g-5p and mml-let-7g-3p_1ss22CT were reversely changed following ischemia. **(D)** Through miRNA-gene-KEGG pathway analysis, mml-let-7g-5p, and mml-let-7g-3p_1ss22CT which share three same target genes such as RB1-inducible coiled-coil 1 (RB1CC1), G-protein subunit γ 5 (GNG5), and chemokine (C-X-C motif) receptor 4 (CXCR4), which was involved in chemokine signaling pathway, AMPK signaling pathway, longevity regulating pathway, ubiquitin mediated proteolysis, fructose, and mannose metabolism.

Some miRNAs were clustered within the genome; as they are transcribed into a polycistron structure, it results in functional synergy. This synergy is indispensable in a complex cellular signaling regulatory network and more effective than a single miRNA. Due to the different regulation of post-transcriptional miRNAs during the maturation process, the expression levels of mature clustered miRNAs differ. By cluster miRNA analysis, we observed that mml-let-7g-5p and mml-let-7g-3p_1ss22CT belonged to one cluster (Table 1 and Figures 4A,B), and that following ischemia their regulation was the opposite: mml-let-7g-5p had high copy numbers in the genome and was upregulated, while mml-let-7g-3p_1ss22CT had low copy numbers and was downregulated after ischemia in the same monkey (Figure 4C). Cytoscape software was used to draw the network diagram of the most significant miRNA: mRNA pairs focusing on mml-let-7g-5p and mml-let-7g-3p_1ss22CT. It has been reported that clustered miRNAs are coordinately transcribed and exhibit similar functions by regulating the same targets (Jiao et al., 2021). Through miRNA-gene-KEGG pathway analysis, we found that mml-let-7g-5p and mml-let-7g-3p_1ss22CT share the same target genes, such as RB1-inducible coiled-coil 1 (RB1CC1), G-protein subunit γ 5 (GNG5), and chemokine (C-X-C motif) receptor 4 (CXCR4), involved in the chemokine signaling pathway, AMPK signaling pathway, longevity regulating pathway, ubiquitin-mediated proteolysis, and fructose, and mannose metabolism (Figure 4D).

### Evolutionary Conservation Analysis of miRNA Sequences and Seed mml-let-7g Sequence

**FIGURE 5:**
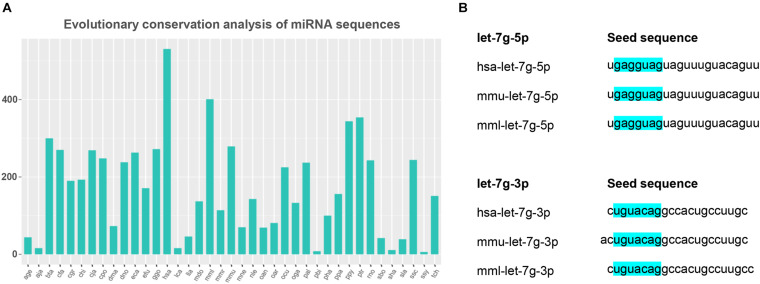
Evolutionary conservation analysis of miRNA sequences and seed mml-let-7g sequence. **(A)** The conservation of miRNA in different species. The horizontal coordinates above represent different species, and the ordinate indicates the number of times the precursor appears in the species. **(B)** The seed sequence of mml-let-7g-5p and mml-let-7g-3p_1ss22CT in human, rhesus monkey, and mouse, which is highlighted in blue.

Based on the analysis of the detected miRNAs, we further analyzed miRNA conservation and performed a statistical analysis of the frequency of these miRNAs in other species. The results showed that miRNAs are highly conserved among species (Figure 5A). Evolutionarily conserved miRNAs usually appear in high copy numbers (Lian et al., 2021). mml-let-7g-5p had high copy numbers, whereas mml-let-7g-3p_1ss22CT had low copy numbers in the rhesus monkey genome, indicating that mml-let-7g-5p is more conserved miRNA than mml-let-7g-3p_1ss22CT.

In animals, miRNA inhibits mRNA expression mainly through incomplete complementary pairing with its target gene. The Watson Crick pairing between the so-called seed sequence (second-7 nt) at the 5′ end of miRNA and the 3′-UTR of the target gene is the most important factor for all miRNA target gene prediction. Twenty-one miRNAs in the let-7 family shared the seed sequence “GAGGTAG.” Human, rhesus monkey, and mouse mml-let-7g-5p share the seed sequence “GAGGTAG,” while the seed sequence of mml-let-7g-3p_1ss22 was “TGTACAG,” indicating that both mml-let-7g-5p and mml-let-7g-3p_1ss22CT were evolutionarily conserved miRNAs (Figure 5B).

## Discussion

This study compared differentially expressed miRNA profiles in the plasma of three rhesus monkeys before and after cerebral ischemia. Of the 2049 miRNAs found, 36 were upregulated, and 76 were downregulated after cerebral ischemia. Further analysis revealed that differentially expressed miRNAs were involved in the regulation of various signaling pathways, including ubiquitin-mediated proteolysis, pathways in cancer, glioma, chronic myeloid leukemia, and chemokine signaling pathways. Through miRNA clustering analysis, we found that mml-let-7g-5p and let-7g-3p_1ss22CT belong to a single cluster and share three target genes: RB1CC1, GNG5, and CXCR4; further, they showed opposite changes following ischemia. These data suggest that clusters of circulating miRNAs of the mml-let-7g family may serve as a therapeutic target for ischemic stroke.

Totally, 112 miRNAs had significantly differential expressions before and after cerebral ischemia, which together with heatmap of DEmiRNAs suggested their potential as biomarkers to distinguish ischemic stroke and provided clues for us to further explore the complex regulatory mechanisms underlying ischemic stroke. To have a general understanding of functions of differentially expressed genes in following cerebral ischemia, we perform GO and KEGG pathway analysis on 112 DEmiRNAs. The enriched GO terms of BP, CC, and MF category referred to nucleus, extracellular exosome, cytoplasm, focal adhesion, and metal ion binding, ATP binding, etc. KEGG analysis showed that genes were significantly enriched in several crucial pathophysiological processes in stroke, such as ubiquitin-mediated proteolysis and signaling pathways in cancer, glioma, chronic myeloid leukemia, and chemokine signaling, etc.

Rfam revealed that rRNA was increased while tRNA was decreased in the plasma of three rhesus monkeys after cerebral ischemia. As it was recently reported that circulating tRNA fragments are a novel biomarker class to distinguish acute stroke subtypes (Nguyen et al., 2020), this topic is worthy of further study. However, the present study focused on miRNA. Many miRNAs are not randomly distributed but linked as a cluster on chromosomes and transcribed as a single polycistronic transcript from genomic DNA. These clustered miRNAs largely appear in metazoan genomes and play pivotal roles in the co-regulation of multiple biological processes. An miRNA gene cluster is composed of two or more related miRNA genes that may target specific mRNAs in the regulatory network. An miRNA duplex contains two “arms,” and the dominant one is the one which usually forms the mature miRNA; the other usually tends to be degraded, and is known as miRNA^∗^ (Medley et al., 2021). In recent years, studies have found that miRNAs and miRNAs^∗^ can co-exist under certain circumstances (Correia de Sousa et al., 2019; Dong et al., 2019; Zhang Z. et al., 2019). However, there is a lack of relevant research on the relationship between them and their functions. Recently, two studies proposed that the two arms of miRNA have synergistic (Dong et al., 2019) or antagonistic functions in various situations (Zhang Z. et al., 2019). Two miRNAs from the same precursor, miR-574-5p and miR-574-3p, were reported to show a special expression pattern, that is, the expression of miR-574-5p was upregulated while that of miR-574-3p was downregulated in the same patient, and this conversion was significantly correlated with the clinical malignancy of the gastric cancer, suggesting that it may be important for the development of gastric cancer (Zhang Z. et al., 2019). As an important regulatory molecule of gene expression, miRNA has been widely studied as a biomarker and therapeutic target in cerebral ischemia (Bulygin et al., 2020; Holmes et al., 2020). However, due to the different species and models used, different miRNAs are often screened out from high-throughput data. However, let-7 family is a common miRNA in these high-throughput data, indicating its core mechanism of ischemic stroke (Bernstein and Rom, 2020; Chen et al., 2020). In our study, we found that two miRNAs from the same precursor, mml-let-7g-5p and mml-let-7g-3p_1ss22CT, had a special expression pattern and similar to the “miR-574 arm transition.” That is, the expression of mml-let-7g-5p was upregulated while that of mml-let-7g-3p_1ss22CT was downregulated in the same monkey. It was previously shown that let-7 family regulate neuroinflammation in various pathologies, including spinal cord injury, multiple sclerosis, ischemic stroke, and Alzheimer’s disease (Gaudet et al., 2018). Let-7g^∗^ reduces the stroke-induced production of proinflammatory cytokines in the mouse brain (Bernstein and Rom, 2020) and protects the blood–brain barrier under neuroinflammatory conditions (Rom et al., 2015), while Let-7g counteracts endothelial dysfunction and ameliorates neurological functions in a mouse ischemia/reperfusion stroke model (Bernstein et al., 2020). Furthermore, whether this conversion was significantly correlated with clinical ischemic stroke and the so-called “mml-let-7g arm transition” was involved in the pathological process needs further study.

In our analysis, mml-let-7g-5p and mml-let-7g-3p_1ss22CT shared the same target genes, such as CXCR4, RB1CC1, and GNG5. CXCR4 is essential for an innate immune system-mediated defense response after cerebral ischemia. CXCR4 distinguishes hematopoietic stem cell-derived monocytes from microglia and reveals monocyte immune responses to experimental stroke (Werner et al., 2020). CXCR4 mimic acts as a soluble chemokine receptor that blocks atherogenic inflammation (Kontos et al., 2020). In addition, CXCR4 and stromal cell-derived factor-1 are regulators of neuronal migration (Shan et al., 2021). Another target, RB1CC1 is essential for autophagy induction, RB1CC1 insufficiency causes neuronal atrophy and is involved in the pathology of Alzheimer’s disease (Chano et al., 2007). Ablation of RB1CC1 results in a progressive loss of neural stem cells and impairment of neuronal differentiation, specifically in the postnatal brain in mice (Wang et al., 2013). Additionally, animals with a dendritic cell-specific deficiency in RB1CC1/Fip200 were protected against encephalomyelitis (Yang et al., 2021). Moreover, knockout of RB1CC1 can make tumor cells more easily killed by T cells and improve the therapeutic effect of immune checkpoint inhibitors in mice. Another target gene, GNG5, controls the number of apical and basal progenitors and alters neuronal migration during cortical development (Ayo-Martin et al., 2020). These data suggest that clusters of circulating miRNAs of the mml-let-7g family may serve as a therapeutic target for ischemic stroke which might target both central nervous system as well as immune system.

The present study aimed to identify miRNAs directly related to ischemic stroke. Thirty-six miRNAs were upregulated and 76 downregulated after cerebral ischemia. Through miRNA clustering analysis we found that mml-let-7g-5p and let-7g-3p_1ss22CT belong to a single cluster, and were conversely regulated following ischemia, and target signaling pathways such as the chemokine signaling, AMPK signaling, and longevity regulating pathways, as well as ubiquitin mediated proteolysis, and fructose and mannose metabolism. They further share three target genes, CXCR4, RB1CC1, and GNG5. These data suggest that circulating mml-let-7g may serve as a therapeutic target for ischemic stroke. This study has several limitations. First, this study is limited with small sample sizes, and is hampered by potential biases. Second, we did not prove mml-let-7g-5p was upregulated while that of mml-let-7g-3p_1ss22CT was downregulated by large sample data. Third, we didn’t identify the targets predicted by these software. Therefore, further studies are needed to determine the mechanism of action of the miRNA candidates involved in ischemic stroke.

## Data Availability Statement

The data presented in the study are deposited in the GEO repository (https://www.ncbi.nlm.nih.gov/geo/query/acc.cgi?acc=GSE182429), accession number (GSE182429).

## Ethics Statement

The animal study was reviewed and approved by Animal Use and Care Board of the Institute of Laboratory Animal Sciences, Capital Medical University.

## Author Contributions

JC, YL, and XJ designed the experiments for the study. HZ, YH, YL, and JF wrote the manuscript. RW, ZH, DW, ZY, and LW performed the experiments and analyzed the data. All authors have read and agreed to the published version of the manuscript.

## Conflict of Interest

The authors declare that the research was conducted in the absence of any commercial or financial relationships that could be construed as a potential conflict of interest.

## Publisher’s Note

All claims expressed in this article are solely those of the authors and do not necessarily represent those of their affiliated organizations, or those of the publisher, the editors and the reviewers. Any product that may be evaluated in this article, or claim that may be made by its manufacturer, is not guaranteed or endorsed by the publisher.
